# Benign Symmetric Lipomatosis Complicated by Severe Obstructive Sleep Apnea During Long-Term Follow-Up: A Case Report

**DOI:** 10.7759/cureus.111761

**Published:** 2026-06-29

**Authors:** Yoshiki Sato, Shintaro Yodogawa, Yurina Wakabayashi, Hiroki Nagayasu, Tsuyoshi Shimo

**Affiliations:** 1 Division of Reconstructive Surgery for Oral and Maxillofacial Region, Department of Human Biology and Pathophysiology, School of Dentistry, Health Sciences University of Hokkaido, Hokkaido, JPN; 2 Division of Oral Maxillofacial Surgery, Department of Human Biology and Pathophysiology, School of Dentistry, Health Sciences University of Hokkaido, Hokkaido, JPN

**Keywords:** benign symmetric lipomatosis, cervical mass, madelung disease, mitochondrial dna, multiple symmetric lipomatosis, nasal cpap, obstructive sleep apnea

## Abstract

Benign symmetric lipomatosis (BSL), also known as Madelung disease, is a rare disorder characterized by symmetric and diffuse deposition of nonencapsulated adipose tissue, predominantly in the neck, shoulders, and upper trunk. Although BSL is often recognized as a slowly progressive cosmetic condition, deep cervical and upper airway involvement may cause clinically significant functional impairment. We report a case of BSL that was reevaluated after referral from a prosthodontic clinic and later complicated by severe obstructive sleep apnea (OSA) during long-term follow-up. An 80-year-old man was referred to our department in 2007 after visiting the prosthodontic clinic because of the detachment of dental prostheses. He had noticed cervical swelling since approximately 1983 and had a history of heavy alcohol consumption and alcoholic hepatitis. Clinical examination and imaging showed symmetric, soft, painless fatty masses in the anterior and posterior neck and bilateral shoulder regions, and BSL was clinically diagnosed. The patient discontinued follow-up but returned in 2015 because of enlargement of the cervical masses, snoring, dyspnea during sleep, daytime sleepiness, and hoarseness. Imaging demonstrated progression of cervical fatty deposition with airway narrowing. Polysomnography revealed severe OSA, with an apnea-hypopnea index of 51.7 events/hour, a 3% oxygen desaturation index of 56.6 events/hour, and a lowest peripheral oxygen saturation of 82%. Nasal continuous positive airway pressure therapy was introduced. Because of the patient's advanced age, the nonencapsulated nature of the lesions, the difficulty of complete resection, and the risks of recurrence and deep bleeding, surgical intervention was not performed. A maternal history of similar cervical swelling prompted screening for representative mitochondrial DNA variants, but no mutations were detected. The patient died of presumed cardiac causes approximately one and a half years later. This case highlights that BSL may progress over decades and cause severe sleep-disordered breathing. Dental and oral surgery clinicians should recognize symmetric cervical swelling as a potential manifestation of BSL and consider airway evaluation when snoring, dyspnea, or daytime sleepiness is present.

## Introduction

Benign symmetric lipomatosis (BSL) is a rare disorder characterized by symmetric and diffuse accumulation of adipose tissue, mainly in the neck, shoulders, and upper trunk. The disease is also known as Madelung disease, multiple symmetric lipomatosis, or Launois-Bensaude syndrome [1-3]. Since Madelung described characteristic cervical fatty deposition in 1888, BSL has been recognized as an uncommon disease associated with abnormal fat metabolism [1,2]. Adipose tissue typically proliferates from the anterior neck to the posterior neck and scapular region, sometimes resulting in a so-called horse-collar deformity or buffalo hump [1-3]. A clinically important feature that distinguishes BSL from solitary lipoma is that the lesions are usually nonencapsulated and poorly demarcated from surrounding tissues [2,3].

BSL predominantly affects middle-aged men and has been reported more frequently in Europe, particularly in Mediterranean regions, whereas reports from Asia remain limited [1,3]. Although the exact epidemiology is difficult to determine because of the rarity of the disease, chronic heavy alcohol consumption, metabolic abnormalities, and familial or genetic backgrounds have been reported as relevant clinical factors [3-5]. The pathogenesis has not been fully clarified; however, chronic heavy alcohol consumption has long been strongly implicated.

Many reported cases are associated with heavy alcohol intake, liver dysfunction, dyslipidemia, and glucose metabolism abnormalities [3-5]. Proposed mechanisms include alcohol-induced impairment of lipolysis and abnormal proliferation of adipocytes derived from brown adipose tissue, but no single mechanism fully explains the disease [3,5]. Recent reviews have broadened this picture beyond alcohol, implicating defective adipocyte differentiation and aberrant intracellular signaling (e.g., Casein Kinase 2-Protein Kinase B-Extracellular Signal-Regulated Kinase activation) in lesion expansion and the high recurrence rate, although no single pathway fully accounts for the disease [5].

Mitochondrial dysfunction has also attracted attention in the pathogenesis of BSL. Associations with mitochondrial DNA abnormalities, particularly the mitochondrial DNA A8344G mutation related to myoclonic epilepsy with ragged-red fibers (MERRFs), have been reported [6,7]. Familial cases have also been described, and in cases with a family history suggestive of maternal inheritance, evaluation for a genetic background may be warranted in addition to assessment of alcohol-related factors [7]. Thus, although BSL appears clinically as excessive fatty deposition, its pathophysiology may involve both metabolic and genetic factors and is not uniform across patients [3,6,7]. More recently, the genetic spectrum has been shown to extend beyond mitochondrial DNA: biallelic variants in Mitofusin 2 (MFN2), which encodes the mitochondrial fusion protein mitofusin-2, have been established as a distinct cause of multiple symmetric lipomatosis that is frequently accompanied by axonal neuropathy, and biallelic LIPE variants have also been reported [5,8].

Clinically, BSL is usually slowly progressive and is often initially regarded as a cosmetic problem. However, when fatty deposition extends into the deep cervical, peripharyngeal, or perilaryngeal regions, it may cause compressive symptoms such as hoarseness, dysphagia, and dyspnea, and it may lead to obstructive sleep apnea (OSA) or even acute upper airway obstruction [3]. Therefore, BSL should not be managed merely as a morphological abnormality; airway evaluation and systemic assessment are important. Although surgical treatment, including lipectomy and liposuction, remains the mainstay of treatment, complete resection is challenging because the lesions lack a capsule and are poorly demarcated. Recurrence is not uncommon, and treatment decisions should consider age, general condition, lesion extent, and the severity of functional impairment [9,10].

Here, we report a case of BSL in which cervical fatty deposition progressed over a long period and was complicated by severe OSA. The patient was reevaluated after presenting for dental care, and disease progression was confirmed by imaging and assessment of sleep-disordered breathing. Conservative management with nasal continuous positive airway pressure (nCPAP) was selected rather than surgical intervention. This case provides useful insight into the long-term course of BSL and its potential airway complications.

## Case presentation

Patient

The patient was an 80-year-old man. In April 2007, he visited the prosthodontic clinic of our hospital with the chief complaint of detachment of maxillary and mandibular dental prostheses. At that time, evaluation of a cervical mass was recommended, and he was referred to our department.

Medical and family history

The patient had been hospitalized for alcoholic hepatitis approximately 40 years earlier. He had a history of heavy daily alcohol consumption from approximately 16-40 years of age. He underwent surgery for a femoral fracture in 1983 and underwent resection of two-thirds of the stomach for gastric cancer in 1990. In 2013, he was diagnosed with prostate cancer, which achieved remission after radiotherapy and chemotherapy. Regarding family history, it was found at the time of reexamination in 2015 that his mother had also had a similar cervical mass.

History of present illness

The patient first noticed cervical swelling around 1983 and consulted the internal medicine department of another university hospital. The swelling was assessed as fatty deposition, and follow-up observation was recommended. However, after several visits, he discontinued follow-up on his own. Thereafter, because he had no clear subjective symptoms, he left the condition untreated. In April 2007, during dental prosthetic treatment, further evaluation of the cervical mass was recommended, and he visited our department. Based on characteristic findings on computed tomography (CT) and ultrasonography, BSL was clinically diagnosed; however, the patient refused additional examinations and again discontinued follow-up. In May 2015, he revisited the prosthodontic clinic because of damage to a maxillary anterior prosthesis. At that time, he noticed enlargement of the cervical mass, snoring during sleep, dyspnea, and daytime sleepiness, and he requested reevaluation in our department.

Clinical findings

At the first visit in 2007, the patient was 156 cm tall, weighed 50.6 kg, and had a body mass index of 20.27 kg/m^2^. His blood pressure was 138/82 mmHg, and peripheral oxygen saturation (SpO_2_) in room air was 98%-99%. Symmetric, soft, painless, fatty masses were observed in the anterior neck, posterior neck, and bilateral shoulder regions. No apparent adhesion to surrounding tissues was detected. Intraorally, mild thickening of the right side of the tongue was observed. The initial extraoral and intraoral findings, together with the panoramic radiographic findings, are shown in Figure 1.

**Figure 1:**
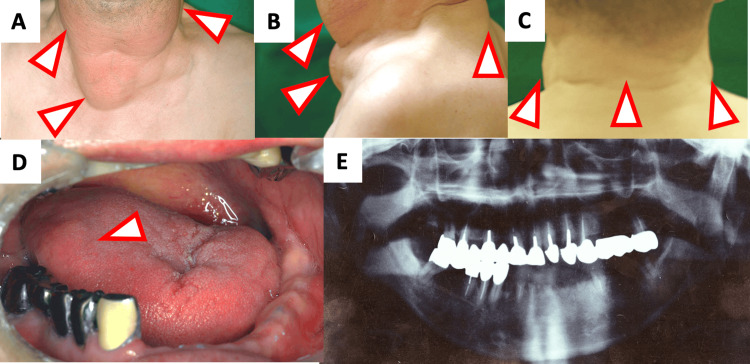
Clinical and radiographic findings at the initial visit in 2007 (A) Frontal extraoral photograph showing symmetric fatty enlargement of the anterior neck (red arrows). (B) Lateral extraoral photograph showing fatty enlargement extending from the cervical region to the shoulder (red arrows). (C) Posterior extraoral photograph showing symmetric fatty enlargement of the posterior neck and bilateral shoulder regions (red arrows). (D) Intraoral photograph showing mild thickening of the right side of the tongue (red arrow). (E) Panoramic radiograph showing no abnormal findings in the jaw bones

Blood tests showed no remarkable abnormalities. At reexamination in 2015, he was 156 cm tall, weighed 51 kg, had a body mass index of 20.7 kg/m^2^, and his blood pressure was 135/84 mmHg. Although his body habitus had not changed substantially, SpO_2_ in room air had decreased to 93%-96%. The fatty masses in the anterior and posterior neck and bilateral shoulder regions were clearly enlarged compared with those observed at the first visit. Hoarseness was also present. The mild thickening of the right side of the tongue had slightly worsened, and saliva retention was observed in the oral cavity. Blood tests showed a tendency toward mild anemia but no other noteworthy abnormalities.

Imaging findings

Panoramic radiography at the first visit in 2007 showed no abnormal findings in the jaw bones. CT demonstrated fatty deposition in the anterior and posterior neck without a discrete encapsulated mass lesion (Figures 2A, 2B), and ultrasonography showed no obvious mass lesion within the fatty tissue. Based on these imaging findings and the characteristic clinical appearance, BSL was clinically diagnosed. CT performed at reexamination in 2015 demonstrated worsening fatty deposition in the anterior neck, posterior neck, and shoulder regions (Figures 2C, 2D). Magnetic resonance imaging (MRI) showed airway narrowing caused by enlarged fatty tissue, suggesting involvement in sleep-disordered breathing (Figures 2E, 2F).

**Figure 2:**
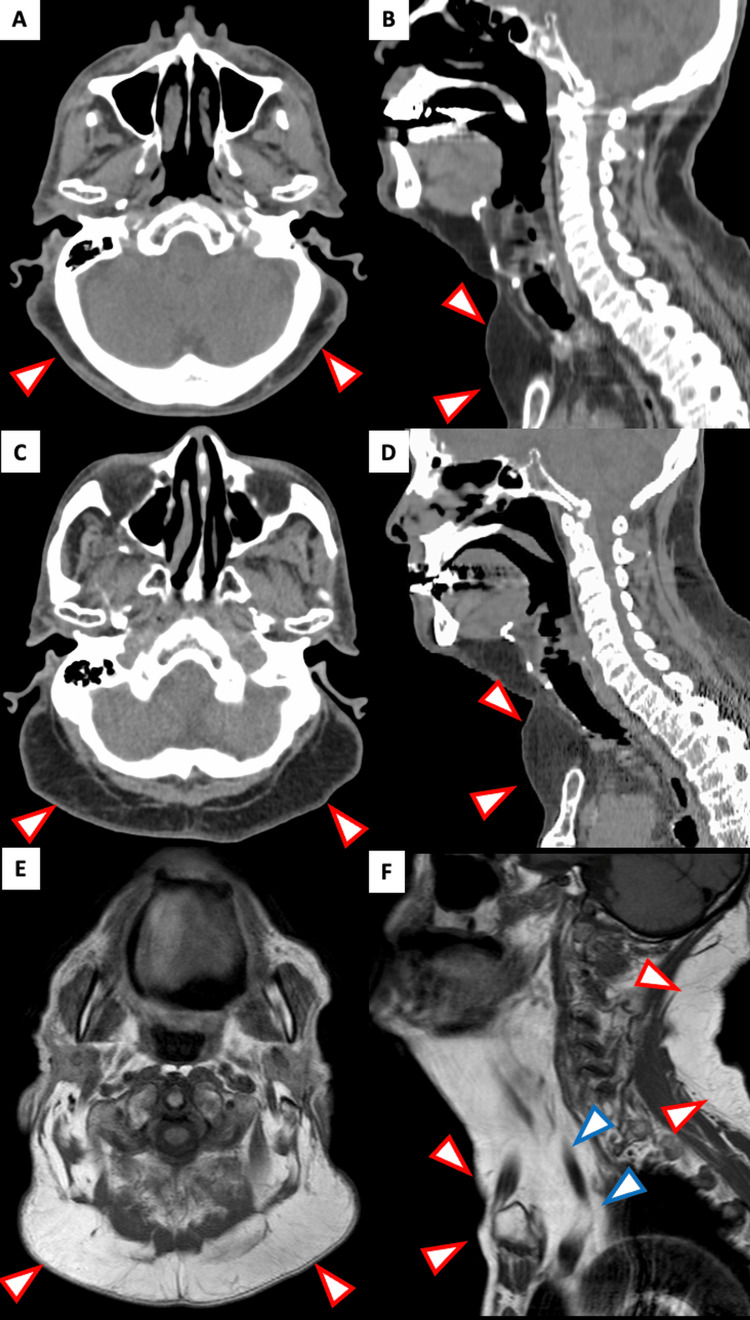
Computed tomography and magnetic resonance imaging findings at the initial visit and reexamination (A,B) Computed tomography images obtained at the initial visit in 2007 showing diffuse fatty deposition in the cervical region without a discrete encapsulated mass lesion (red arrows). (C,D) Computed tomography images obtained at reexamination in 2015, showing progression of fatty deposition in the anterior and posterior neck regions (red arrows). (E,F) Magnetic resonance imaging obtained in 2015 showing enlarged cervical fatty tissue (red arrows) and airway narrowing (blue arrows)

Clinical diagnosis

On the basis of symmetric and diffuse adipose tissue deposition centered in the cervical region and the imaging findings, the patient was clinically diagnosed with BSL.

Treatment and clinical course

At the first visit in 2007, the patient had few subjective symptoms and did not wish to undergo further examination or treatment. Therefore, only radiography, CT, ultrasonography, and blood testing were performed, after which he discontinued follow-up. At reexamination in 2015, enlargement of cervical fatty tissue and worsening sleep-related symptoms suggested OSA, and the patient was referred to the otolaryngology department of another university hospital. Overnight polysomnography showed an apnea-hypopnea index (AHI) of 51.7 events/hour, a 3% oxygen desaturation index of 56.6 events/hour, and a lowest oxygen saturation (SpO₂) of 82%, leading to a diagnosis of severe OSA (Table 1).

**Table 1:** Summary of polysomnography findings in 2015 The polysomnography findings were consistent with severe obstructive sleep apnea ODI: oxygen desaturation index; SpO₂: peripheral oxygen saturation

Parameter	Result
Apnea-hypopnea index	51.7 events/hour
3% oxygen desaturation index (3% ODI)	56.6 events/hour
Lowest oxygen saturation (SpO₂)	82%
Diagnosis	Severe obstructive sleep apnea

nCPAP therapy was promptly introduced. Regarding treatment strategy, surgery was not performed after discussion with the collaborating otolaryngology department because of the patient's advanced age, the nonencapsulated nature of the lesions, the difficulty of complete resection, the high recurrence rate reported after fat-reduction surgery, and the potential risk of deep bleeding associated with liposuction or lipectomy. Instead, conservative observation under nCPAP therapy was selected. Because his mother reportedly had a similar cervical mass, mitochondrial DNA abnormalities were suspected. Screening was performed for m.8344A>G, m.8356T>C, m.8363G>A, m.1555A>G, m.3271T>C, m.8993T>G/C, m.9176T>C, m.11778G>A, and m.13513G>A variants; however, none of these mutations were detected (Figure 3).

**Figure 3:**
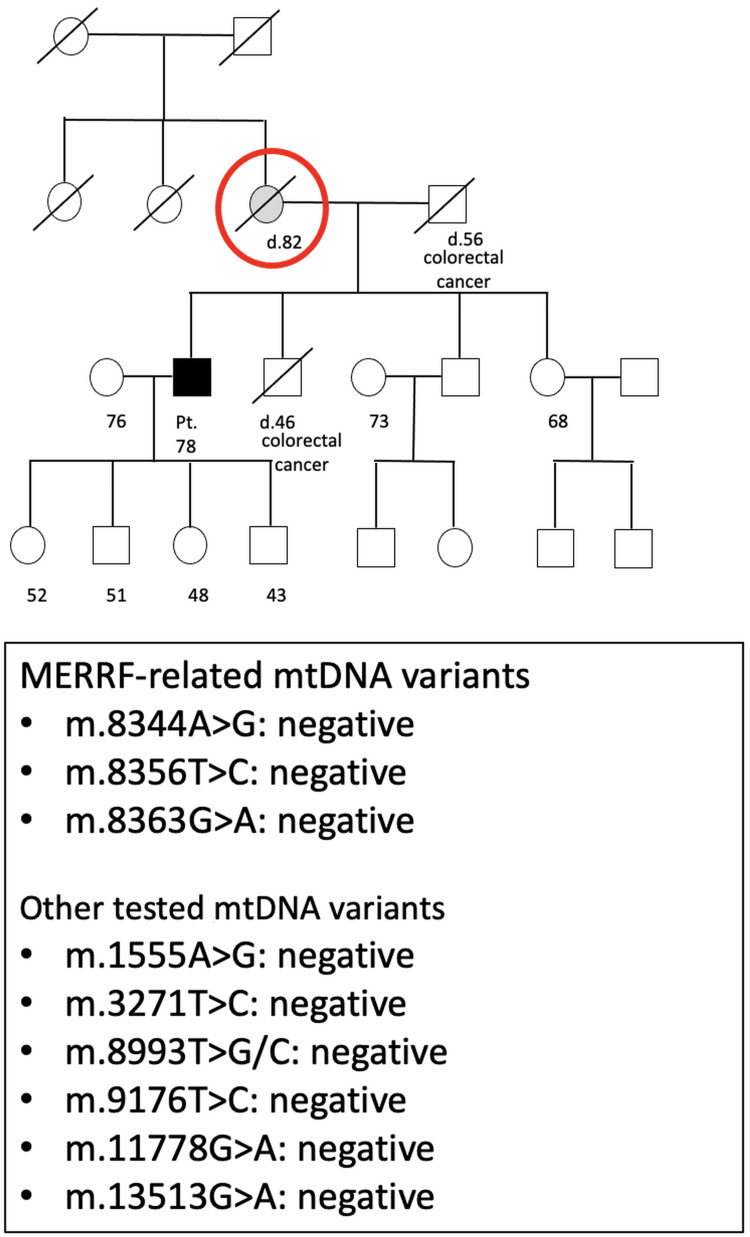
Family history and mitochondrial DNA analysis The pedigree shows the patient’s family history. The patient reported that his mother had a similar cervical mass, and colorectal cancer was noted in two relatives. Mitochondrial DNA analysis showed no detectable mutations in the tested MERRF-related variants, including m.8344A>G, m.8356T>C, and m.8363G>A, or in the other tested variants, including m.1555A>G, m.3271T>C, m.8993T>G/C, m.9176T>C, m.11778G>A, and m.13513G>A. The red circle indicates the patient’s mother, who reportedly had a similar cervical mass MERRF: myoclonic epilepsy with ragged-red fibers; mtDNA: mitochondrial DNA

Thereafter, follow-up was continued in collaboration with the otolaryngology department, with attention to airway obstruction caused by further enlargement of fatty tissue. However, the patient died at home of presumed cardiac causes, one and a half years later.

## Discussion

BSL is a rare disorder characterized by symmetric, nonencapsulated adipose tissue deposition mainly in the neck, shoulders, and upper trunk. It is also known as Madelung disease, multiple symmetric lipomatosis, or Launois-Bensaude syndrome [1-3]. BSL is most common in men aged 30-60 years, and the male-to-female ratio has been reported to be approximately 15:1 to 30:1 [1]. Although our patient was elderly at 80 years of age, he exhibited typical clinical and imaging features of BSL, including symmetric and diffuse fatty deposition from the neck to the shoulders, fat-density lesions on CT, and homogeneous high signal intensity on T1-weighted MRI comparable to that of surrounding adipose tissue [1-3]. Several anatomical classifications of BSL have been proposed. According to the classification of Donhauser et al., the cervical and shoulder-girdle distribution in our patient corresponded to a mixed type I (horse-collar) and type II (pseudoathletic) pattern, whereas the lesions did not correspond to the clinical types described by Carlsen and Thomsen [11,12]. Because more than one distribution type frequently coexists in individual patients, such schemes are useful mainly for descriptive purposes rather than for predicting clinical behavior [11,12].

The differential diagnosis of BSL includes simple obesity, solitary or multiple lipomas, liposarcoma, Cushingoid fat deposition, and other causes of cervical or upper body soft-tissue enlargement. In the present case, simple obesity was unlikely because the fatty enlargement was regionally localized to the neck and shoulder areas rather than generalized. Solitary lipoma and liposarcoma were also considered less likely because imaging showed diffuse, symmetric, nonencapsulated fatty deposition without a discrete mass lesion or suspicious invasive features. Although endocrine or metabolic causes of abnormal fat distribution should also be considered, the characteristic symmetric distribution, long-term progression, family history, and imaging findings supported the clinical diagnosis of BSL.

The pathogenesis of BSL remains incompletely understood, but chronic heavy alcohol consumption has long been implicated. Heavy alcohol intake has been reported in 60%-90% of cases [3,4]. Alcohol may impair catecholamine-dependent lipolysis in adipocytes and promote abnormal proliferation or reduced lipolysis of adipocytes derived from brown adipose tissue [3,4]. Our patient had a history of long-term heavy alcohol consumption from approximately 16-40 years of age and previous alcoholic hepatitis; these factors may have contributed to the onset and progression of BSL. However, fatty deposition gradually progressed even after long-term cessation of heavy drinking, suggesting that alcohol alone could not fully explain the disease course and that other metabolic or genetic factors might also have been involved [3,4]. This interpretation is consistent with recent pathogenesis reviews, which regard alcohol as a facilitator rather than a sole cause and emphasize the contributions of impaired mitochondrial function and dysregulated adipocyte differentiation to lesion formation [5].

Associations between BSL and mitochondrial dysfunction have also been reported, particularly with the mitochondrial DNA A8344G mutation related to MERRF [6,7]. Mitochondrial genetic abnormalities have been detected in a subset of patients, and A8344G is one of the representative variants [6,7]. Familial cases have also been reported, and screening for mitochondrial DNA abnormalities may be meaningful in patients with a family history suggestive of maternal inheritance [7]. In the present case, the patient reported that his mother had a similar cervical mass, and therefore, representative mitochondrial DNA variants were screened. No mutation was detected among the tested variants. Thus, this case may be regarded as BSL without detectable representative mitochondrial DNA mutations; however, because the molecular pathogenesis of BSL is heterogeneous, negative screening results do not completely exclude a genetic background [6,7]. This is especially relevant because the recognized genetic causes of multiple symmetric lipomatosis now extend to nuclear genes, including biallelic MFN2 mutations [8] and, more recently, LIPE variants [5], which are not captured by targeted mitochondrial DNA screening. Accordingly, our negative mitochondrial DNA results do not exclude an underlying genetic predisposition, and broader genetic evaluation such as whole-exome sequencing may be warranted in familial or syndromic presentations [5,8].

BSL is generally slowly progressive and is often recognized as a cosmetic condition. However, when the lesions extend into the deep cervical and perilaryngeal regions, they may cause hoarseness, dysphagia, dyspnea, OSA, or acute upper airway obstruction [3]. Reported cases of BSL or Madelung disease associated with OSA or significant upper airway obstruction are summarized in Table 2 [13-20].

**Table 2:** Reported cases of benign symmetric lipomatosis associated with obstructive sleep apnea or significant upper airway obstruction The present case is included for comparison with previously reported cases NR: not reported; SAS: sleep apnea syndrome; OSA: obstructive sleep apnea; CPAP: continuous positive airway pressure; BiPAP: bilevel positive airway pressure; nCPAP: nasal continuous positive airway pressure

Study	Age	Sex	Distribution	OSA severity	Treatment	Outcome
Truy et al. [13]	NR	NR	NR	SAS	NR	NR
Mégevand et al. [14]	NR	NR	NR	SAS	CPAP	Improved
Ali and Kishore [15]	58	M	Head and neck	OSA	Surgery	Improved
Fonseca et al. [16]	42	M	Neck/shoulder	Severe OSA	BiPAP	Improved
Pinto et al. [17]	56	M	NR	Severe OSA	CPAP → pharyngoplasty	Improved
Segsarnviriya and Chirakalwasan [18]	51	M	Neck/upper trunk	Severe OSA	CPAP	Improved
Tsilivigkos et al. [19]	56	M	Neck	Upper airway obstruction	Surgery + tracheostomy	Improved
Fukuda et al. [20]	58	M	Neck (circumferential)	Upper airway obstruction	Staged surgical resection	Improved
Present case	80	M	Neck/shoulders	Severe OSA	nCPAP	Controlled

Compared with previously reported cases summarized in Table 2, the present case is notable for the patient’s advanced age and the long duration of clinical follow-up. In previously reported cases with available age data, patients were 42-58 years old, whereas the present patient was 80 years old. In addition, the present case objectively documented severe OSA by polysomnography, with an AHI of 51.7 events/hour, and demonstrated long-term progression of cervical fatty deposition on follow-up imaging. These features highlight the clinical relevance of recognizing airway compromise during long-term follow-up of BSL, particularly in elderly patients.

In the present case, no obvious respiratory symptoms were observed at the first visit in 2007. However, by the 2015 reexamination, enlargement of cervical fatty deposition was accompanied by snoring, dyspnea, and daytime sleepiness. MRI showed airway narrowing, and polysomnography confirmed severe OSA, as summarized in Table 1. This clinical course is important because it shows that BSL is not merely a morphological abnormality but may progress over time to cause functional airway impairment [3,13-20].

Surgical treatment, including lipectomy and liposuction, remains the main therapeutic approach for BSL. However, because the lesions lack a capsule and are poorly demarcated from surrounding tissue, complete resection is difficult and recurrence is common [9,10]. Recent clinical analyses have reported that surgery is performed in more than 70% of patients, whereas postoperative recurrence occurs in approximately 40% of cases; age, body mass index, alcohol history, and comorbidities may influence recurrence [9]. Alcohol abstinence may help suppress lesion progression, but it does not reliably reduce existing fatty deposition [3,10]. In our patient, avoidance of surgical intervention was considered appropriate because of his advanced age, the potential invasiveness and bleeding risk of surgery for deep cervical lesions, and the feasibility of conservative management after nCPAP was introduced for severe OSA. On the other hand, if further enlargement had caused progressive dyspnea, recurrent laryngeal nerve dysfunction, dysphagia, or superior vena cava syndrome, fat-reduction surgery or liposuction would have required reconsideration [9,10].

The clinical significance of this case lies in three points. First, BSL was reevaluated after a dental visit. Second, progression of cervical fatty deposition during long-term follow-up was associated with the development of severe OSA. Third, conservative management with nCPAP, rather than surgery, was selected. In dental and oral surgery practice, symmetric cervical swelling should not be overlooked as simple obesity or localized fatty deposition, and BSL should be considered in the differential diagnosis.

When symptoms such as snoring, daytime sleepiness, or dyspnea are present, airway evaluation and assessment for sleep-disordered breathing should be actively performed in collaboration with otolaryngologists and sleep medicine specialists [3,13-20]. Limitations of this case include the absence of histopathological confirmation, the limited scope of genetic testing, and the inability to directly prove a causal relationship between the patient's death from presumed cardiac causes one and a half years later and OSA.

Nevertheless, this case is informative because it demonstrates that BSL may progress over a long period and cause functional airway compromise.

## Conclusions

We reported a case of BSL that progressed over a long period and was complicated by severe OSA. The disease was reevaluated after dental care, and the clinical progression was supported by imaging and polysomnography. Because of the patient's advanced age, the nonencapsulated nature of the lesions, and the potential risks of surgery, nCPAP therapy and conservative observation were selected. Clinicians should recognize that BSL can cause clinically significant airway obstruction during long-term follow-up. In patients with symmetric cervical fatty deposition and symptoms such as snoring, dyspnea, or daytime sleepiness, multidisciplinary airway assessment should be considered.
